# Evaluating the Accuracy of Google Translate for Diabetes Education Material

**DOI:** 10.2196/diabetes.5848

**Published:** 2016-06-28

**Authors:** Xuewei Chen, Sandra Acosta, Adam Etheridge Barry

**Affiliations:** 1 Transdisciplinary Center for Health Equity Research Department of Health and Kinesiology Texas A&M University College Station, TX United States; 2 Texas A&M University Department of Educational Psychology Texas A&M University College Station, TX United States; 3 Texas A&M University Department of Health and Kinesiology Texas A&M University College Station, TX United States

**Keywords:** health literacy, health education, health communication, language translation, diabetes, machine translation, human interpreter, translator

## Abstract

**Background:**

Approximately 21% of the US population speaks a language other than English at home; many of these individuals cannot effectively communicate in English. Hispanic and Chinese Americans, in particular, are the two largest minority groups having low health literacy in the United States. Fortunately, machine-generated translations represent a novel tool that non-English speakers can use to receive and relay health education information when human interpreters are not available.

**Objective:**

The purpose of this study was to evaluate the accuracy of the Google Translate website when translating health information from English to Spanish and English to Chinese.

**Methods:**

The pamphlet, “You are the heart of your family…take care of it,” is a health education sheet for diabetes patients that outlines six tips for behavior change. Two professional translators translated the original English sentences into Spanish and Chinese. We recruited 6 certified translators (3 Spanish and 3 Chinese) to conduct blinded evaluations of the following versions: (1) sentences translated by Google Translate, and (2) sentences translated by a professional human translator. Evaluators rated the sentences on four scales: fluency, adequacy, meaning, and severity. We performed descriptive analysis to examine differences between these two versions.

**Results:**

Cronbach's alpha values exhibited high degrees of agreement on the rating outcome of both evaluator groups: .919 for the Spanish evaluators and .972 for the Chinese evaluators. The readability of the sentences in this study ranged from 2.8 to 9.0 (mean 5.4, SD 2.7). The correlation coefficients between the grade level and translation accuracy for all sentences translated by Google were negative (eg, *r*_Meaning_=-.660), which indicates that Google provided accurate translation for simple sentences. However, the likelihood of incorrect translation increased when the original English sentences required higher grade levels to comprehend. The Chinese human translator provided more accurate translation compared to Google. The Spanish human translator, on the other hand, did not provide a significantly better translation compared to Google.

**Conclusion:**

Google produced a more accurate translation from English to Spanish than English to Chinese. Some sentences translated by Google from English to Chinese exhibit the potential to result in delayed patient care. We recommend continuous training and credential practice standards for professional medical translators to enhance patient safety as well as providing health education information in multiple languages.

## Introduction

Health promotion and education material from health organizations, as well as mass media, are primarily written and delivered in English. While public health professionals are working to produce more health content and material in other languages, current availability remains limited [1,2]. For patients and caregivers with limited English proficiency (LEP), this lack of health information in their native language can be especially burdensome and represents an important public health issue.

LEP individuals, defined as any person age 5 and older who speaks English “less than very well” [3], represent a vulnerable population that experiences significant health disparities in the United States [4]. Compared to the native English-speaking population, LEP individuals are less likely to receive and understand health information or correctly interpret health education messages [5].

As a result of their lack of comprehension and/or misinterpretation, LEP individuals (1) spend extra time and money seeking and using health care services, (2) have unsatisfactory experiences with health care providers, (3) make inappropriate health decisions, (4) have limited access and use of preventive health care services, (5) are more challenging to recruit into health education programs, (6) take incorrect dosages of medication, and (7) have worse health status [6-11]. These issues become increasingly important to address as the LEP population in the United States continues to steadily grow. According to the US Census Bureau, approximately 21% of the US population (60.6 million) speaks a language other than English at home [12]. Moreover, among foreign-born US adults, nearly three out of four speak limited English or do not speak English at all [13].

Machine-generated translations represent a novel tool that non-English speakers can use to receive and relay health education information when human interpreters are not available. With the proliferation of online technology, 87% of US adults had access to the Internet in 2014, compared to 43% in 2000 [14]. Moreover, the Internet is becoming increasingly prevalent among minority populations [15]. Perry and Mittelmark [16] contend that digital translation tools “offer substantial time and cost saving…can thus be used not only to immediately collect information when the content is not translated, but also to immediately deliver information generated in one language to speakers of other languages” (p. 199). However, miscommunication through translation is possible given that words often have different meanings depending on the context in which they are used [16].

Khanna et al [17] compared English-to-Spanish translation accuracy between Google and human translators for patient education texts, reporting that Google Translate made more errors than human translators and people preferred the human translation for complex sentences. Similarly, Sharif and Tse [18] reported an overall 50% error rate for medicine labels translated from English to Spanish by computer programs. Google Translate has also exhibited a high rate of translation errors when translating content on state and national public health websites from English to Chinese [19]. However, to date, we are unaware of any studies evaluating the outputs of a machine translation tool when translating from English to multiple languages drawn from health education material on diabetes. Therefore, it is critical to identify and evaluate available translation tools for helping LEP speakers of different languages understand English health education material.

The purpose of this pilot study was to evaluate the feasibility and accuracy of the Google Translate website as a tool to help LEP persons understand chronic condition management and prevention strategies. Specifically, Google Translate was used for translating a diabetes patient education pamphlet, distributed by the National Diabetes Education program, from English to Spanish and English to Chinese (Mandarin). We chose to focus on Spanish and Chinese for several reasons. First, Spanish and Chinese speakers are more likely to have limited English proficiency. In the United States, approximately 47% of the foreign-born population from Mexico speaks English “not well” or “not at all,” and 32% of the foreign-born population from China speaks English “not well” or “not at all” [13]. Second, among the LEP population, Chinese (68%) respondents exhibit low health literacy, followed by Latinos (45%) [20]. Third, the prevalence of diabetes is rapidly increasing among Hispanic and Chinese Americans [21]. The following research questions guided this investigation:

1. What is the accuracy of Google translations of written sentences from English to Spanish, when compared to professional human translators?

2. What is the accuracy of Google translations of written sentences from English to Chinese, when compared to professional human translators?

3. Can Google Translate be a safe and accurate alternative to human interpreters for providing translation services on health education materials to LEP patients?

## Methods

### Materials to be Translated

We chose a freely accessible diabetes patient education pamphlet as a heuristic example for evaluating the accuracy of machine translation devices. The pamphlet, “You are the heart of your family…take care of it,” is published by the National Institutes of Health and the Centers for Disease Control and Prevention and distributed by the National Diabetes Education Program. This pamphlet includes six written sentences as behavior change suggestions for managing diabetes and three recommended questions for patients to ask their clinicians. This paper examines the accuracy of Google Translate when translating the six written diabetes prevention and management strategies to determine the differences between machine and human translators, which could be used to direct further research. This study was approved by the Texas A&M University Institutional Review Board.

### Procedures

**Figure 1:**
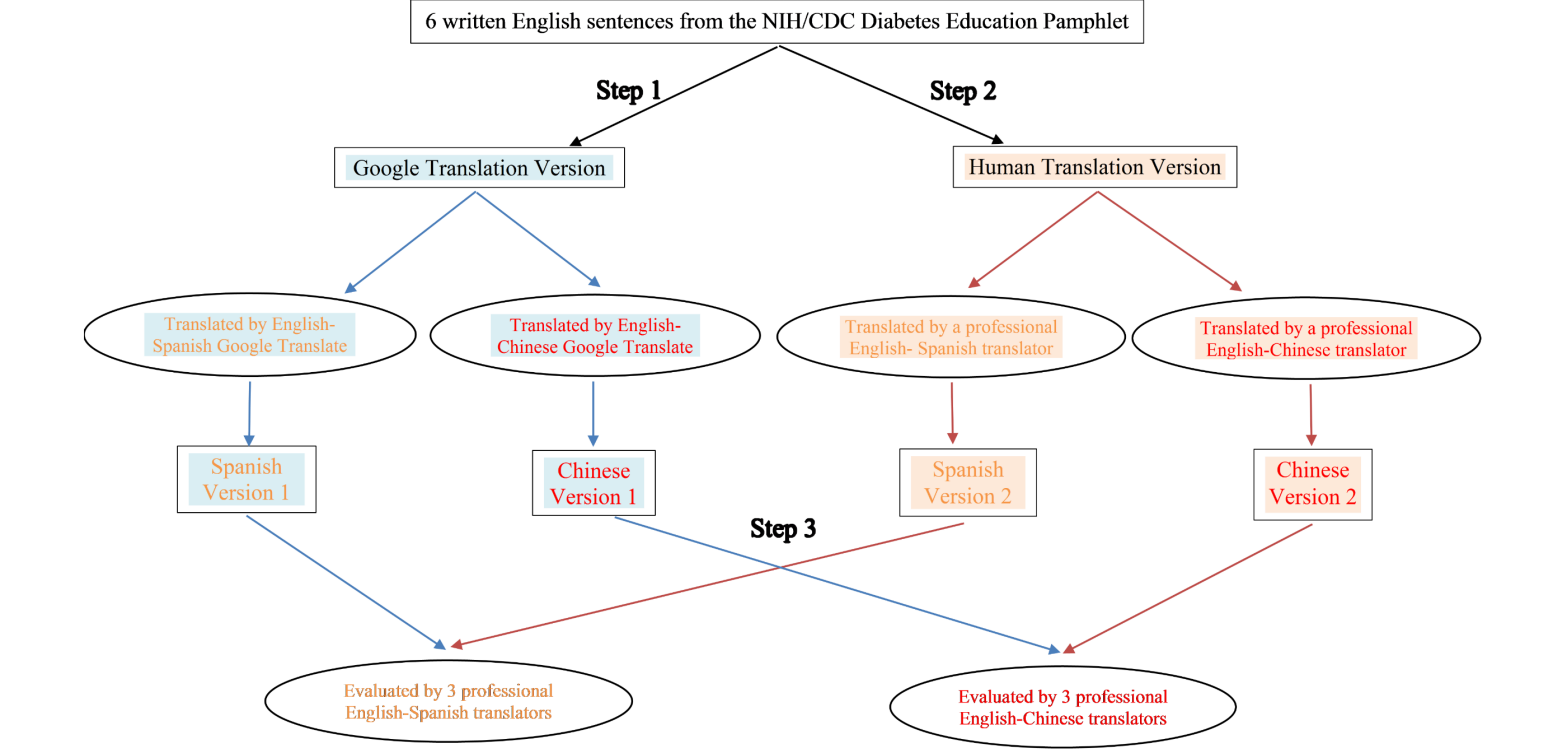
Study procedure.

Following are the overall procedures (see Figure 1) used throughout this investigation.

#### Step 1. Google Translate

We used Google Translate, a free language translation website that instantly translates text and Web pages, to translate the six sentences from English into both Spanish and Chinese.

#### Step 2. Human Translate

Two professional medical translators translated the original English pamphlet into Spanish and Chinese, respectively. Both were American Translators Association (ATA)‒certified translators (one certified in English to Spanish and the other in English to Chinese). The ATA website lists all the certified translators’ contact information. We approached both translators as regular customers seeking translation services. We did not inform them that their translation product would be evaluated. We sent the original English materials to them by email; they returned the translated sentences in Microsoft Word to us by email. All human translation services were paid for based on quotes provided by the individual translators.

#### Step 3. Evaluation

After having the materials translated, we separately recruited 6 ATA-certified translators to evaluate the two translation versions (one by Google Translate and the other one by professional human translators). The two translators who provided the human translation versions did not serve as evaluators, nor were they aware we would have evaluators evaluate their translations. Evaluators were also approached via email. We randomly sent invitation emails to 12 English-Spanish translators and 12 English-Chinese translators. We emailed the survey package to the first 6 translators (3 Spanish and 3 Chinese respectively) who accepted our study invitation. They became the evaluators for this study. Each evaluator received US $15 after submitting the evaluation survey package via email.

### Survey Package

To minimize bias, we did not inform the evaluators which version was created by a human or a machine; instead, we marked the products as version 1 (sentences translated by Google) and version 2 (sentences translated by a human). The survey package contained three separate Microsoft Word documents: (1) an evaluation rubric, (2) translation version 1, and (3) translation version 2. Both versions consisted of six written sentences with the original English sentences listed first, followed by the translated sentences (Spanish or Chinese). We asked the evaluators to score each of the translated sentences based on the included evaluation rubric.

### Evaluation Rubric

Our evaluation rubric, which was adapted from Khana et al [17], asked evaluators to rate the translation sentences based on Fluency, Adequacy, Meaning, and Severity on a 5-point scale (1 indicating low accuracy and 5 indicating high accuracy). The Fluency and Adequacy evaluations are standard domains for assessing machine translation accuracy [22]. The Fluency domain evaluated readability, grammar, and understandability. The Adequacy domain evaluated how much of the original information had been preserved. The Meaning domain assessed whether the translation product had the same meaning as the original sentence. If a translation product added extra information, it could still receive a high Adequacy score as long as it included all the original information. The Meaning score, however, could identify misleading added information [23]. The evaluators also rated the Severity domain, which provided insight into the degree of negative impact on the patient’s health outcome. The detailed evaluation rubric (see Table 1) defined the different categories for each domain.

**Table 1:** Evaluation rubric.

	Fluency	Adequacy	Meaning	Severity
1	No fluency; no appreciable grammar, not understandable	0% of information conveyed from the original	Totally different meaning from the original	Dangerous to patient
2	Marginal fluency; several grammatical errors	25% of information conveyed from the original	Misleading information added/omitted compared to the original	Impairs care in some way
3	Good fluency; several grammatical errors, understandable	50% of information conveyed from the original	Partially the same meaning as the original	Delays necessary care
4	Excellent fluency; few grammatical errors	75% of information conveyed from the original	Almost the same meaning as the original	Unclear effect on patient care
5	Perfect fluency; like reading a newspaper	100% of information conveyed from the original	Same meaning as the original	No effect on patient care

### Data Analysis

We used Cronbach's alpha to assess the degree of agreement among the evaluators. We calculated two sets of means to represent the scores in each of the four domains (ie, Fluency, Adequacy, Meaning, and Severity) from the Chinese and Spanish evaluator groups. We performed descriptive analysis to capture the trend of change from sentence to sentence. Pearson correlation coefficients were also reported to examine the relationship between translation accuracy and the readability of the original English sentences. The readability statistics were generated using Microsoft Word’s Flesch-Kincaid Grade Level, which assesses the degree of difficulty for readers to understand a certain sentence or paragraph [24].

To examine the correlational patterns in the data, we considered using multivariate analysis of variance (MANOVA) for investigating whether there was a statistically significant difference between Google and the professional translators with regard to the translation accuracy. MANOVA allows for the comparison of two groups on these four translation accuracy domains simultaneously [25]. However, *P* values are closely dependent on sample size [26]. Thus, such significance testing is not appropriate in this study due to our small sample size (N=6) and the violation of MANOVA assumptions (eg, normality and homogeneity of variance). Therefore, instead of conducting MANOVA, we presented two sets of graphs to visually compare the translation accuracy between Google and human.

## Results

### Inter-rater Reliability

Cronbach's alpha was used to assess the rating reliability across each evaluator. Cronbach's alpha values exhibited high degrees of agreement on the rating outcome of both evaluator groups: .919 for the Spanish evaluators and .972 for the Chinese evaluators.

### Grade Level and Correlations With Accuracy Scores

Table 2 shows the Flesch-Kincaid Grade Level for all six original English sentences. The Flesch-Kincaid readability test rates text on a US school grade level [24]. The readability of the sentences in this study ranged from 2.8 to 9.0 (mean 5.4, SD 2.7). Shorter sentences with simpler vocabulary received lower scores (eg, grade level=2.9 for S4), and longer sentences containing more medical terms received higher scores (eg, grade level=9.0 for S6).

**Table 2:** Flesch-Kincaid grade level.

Original sentences	Flesch-Kincaid grade level
S1. Eat more fruits, vegetables, beans, and whole grains.	3.7
S2. Eat tasty foods that have less salt, saturated fat, and trans fat.	4.8
S3. Get at least 30 minutes of physical activity on most days or every day. Physical activity helps you keep a healthy weight.	8.5
S4. Stop smoking.	2.8
S5. Take medicines the way your doctor tells you.	3.7
S6. Ask your doctor about taking medicine to protect your heart, such as aspirin or a statin.	9.0
Mean (SD)	5.4 (2.7)

The higher grade level indicates that the text is more difficult for readers to understand. As shown in Table 3, the correlation coefficients between the grade level and translation accuracy for all sentences translated by Google (both Spanish and Chinese) were negative. None of the correlation coefficients was statistically significant at alpha <.05 level due to the small sample size in our study (N=6). However, these negative associations were relatively strong, especially among the Chinese Google group (eg, *r*_Meaning_=-.660). For the sentences translated by the professional human translators, there was only one negative correlation between grade level and translation accuracy scores (*r*_Fluency_=-.447). The correlation coefficients between the grade level and translation accuracy scores show that Google provides more accurate translation for easier sentences but produces more translation errors for more complex sentences. However, the accuracy scores of translated sentences provided by human translators had no strong negative associations with the readability level of the sentences.

**Table 3:** Correlations between grade level and translation accuracy.

Domains	Flesch-Kincaid grade level				
	Spanish		Chinese		
	Google	Human		Google	Human
Fluency	-.374	-.447		-.373	.679
Adequacy	-.162	.120		-.371	.481
Meaning	-.259	.207		-.660	.481
Severity	-.097	.341		-.469	^a^

^a^Correlation coefficient cannot be computed because all sentences translated by the Chinese human translator had a constant severity score (Severity=5).

### Spanish Translation: Google Versus Human

As shown in Table 4, in the Fluency domain, all sentences translated by Google had at least good fluency (Fluency≥3). All sentences translated by the Spanish human translator had excellent or perfect fluency.

In the Adequacy domain, most sentences from both versions conveyed more than 75% of the original information. One sentence translated by the Spanish human translator (S5) conveyed 50% of the original information (Adequacy=3).

In the Meaning domain, similarly, all sentences from both versions had almost the same meaning as the original information. However, S5 translated by the Spanish human translators had partially the same meaning as the original sentence (Meaning=3).

In the Severity domain, all evaluators agreed that S5 translated by Google had an unclear effect on patient care (Severity=4). That same sentence translated by the Spanish human translator delayed necessary patient care (Severity=3).

**Table 4:** Spanish Google versus human.

Original sentences	Google				Human			
	Fluency	Adequacy	Meaning	Severity	Fluency	Adequacy	Meaning	Severity
S1. Eat more fruits, vegetables, beans, and whole grains.								
	4.67	5	5	5	4.33	5	4.67	5
S2. Eat tasty foods that have less salt, saturated fat, and trans fat.								
	3	4.67	4.33	5	4.67	4.67	4.67	4.67
S3. Get at least 30 minutes of physical activity on most days or every day. Physical activity helps you keep a healthy weight.								
	3	4.33	4	4.67	4.67	5	4.67	5
S4. Stop smoking.								
	5	5	5	5	5	5	5	5
S5. Take medicines the way your doctor tells you.								
	4.33	4.33	4.33	4	4.67	3	3	3
S6. Ask your doctor about taking medicine to protect your heart, such as aspirin or a statin.								
	4.67	5	5	4.67	4.33	4.33	4.67	5

### Chinese Translation: Google Versus Human

As shown in Table 5, in the Fluency domain, S2, S3, and S5 translated by Google had marginal or no fluency (Fluency≤2). Every evaluator agreed that S5 was not understandable. All sentences translated by the Chinese human translator had excellent or perfect fluency.

In the Adequacy domain, S5 translated by Google conveyed less than 50% of the original information (Adequacy<3). All sentences translated by the Chinese human translator conveyed almost 100% of the original information.

In the Meaning domain, S3 and S5 translated by Google had less than partially the same meaning as the original information (Meaning<3). All sentences translated by the Chinese human translator had the same or almost the same meaning as the original ones.

In the Severity domain, S5 and S6 translated by Google delayed necessary care for patients (Severity<3). All sentences translated by the Chinese human translator had no effect on patient care (Severity *=* 5).

**Table 5:** Chinese Google versus human.

Original sentences	Google				Human			
	Fluency	Adequacy	Meaning	Severity	Fluency	Adequacy	Meaning	Severity
S1. Eat more fruits, vegetables, beans, and whole grains.								
	4.67	5	4.67	5	5	5	5	5
S2. Eat tasty foods that have less salt, saturated fat, and trans fat.								
	2	4.33	3.67	4.67	4.67	5	5	5
S3. Get at least 30 minutes of physical activity on most days or every day. Physical activity helps you keep a healthy weight.								
	1.67	3.67	2.67	4	5	5	5	5
S4. Stop smoking.								
	5	5	5	5	4.67	4.67	4.67	5
S5. Take medicines the way your doctor tells you.								
	1	2.67	2.67	2.33	4.67	5	5	5
S6. Ask your doctor about taking medicine to protect your heart, such as aspirin or a statin.								
	3	3.67	3	2.33	5	5	5	5

### Visually Comparing Google and Human Versions

**Figure 2:**
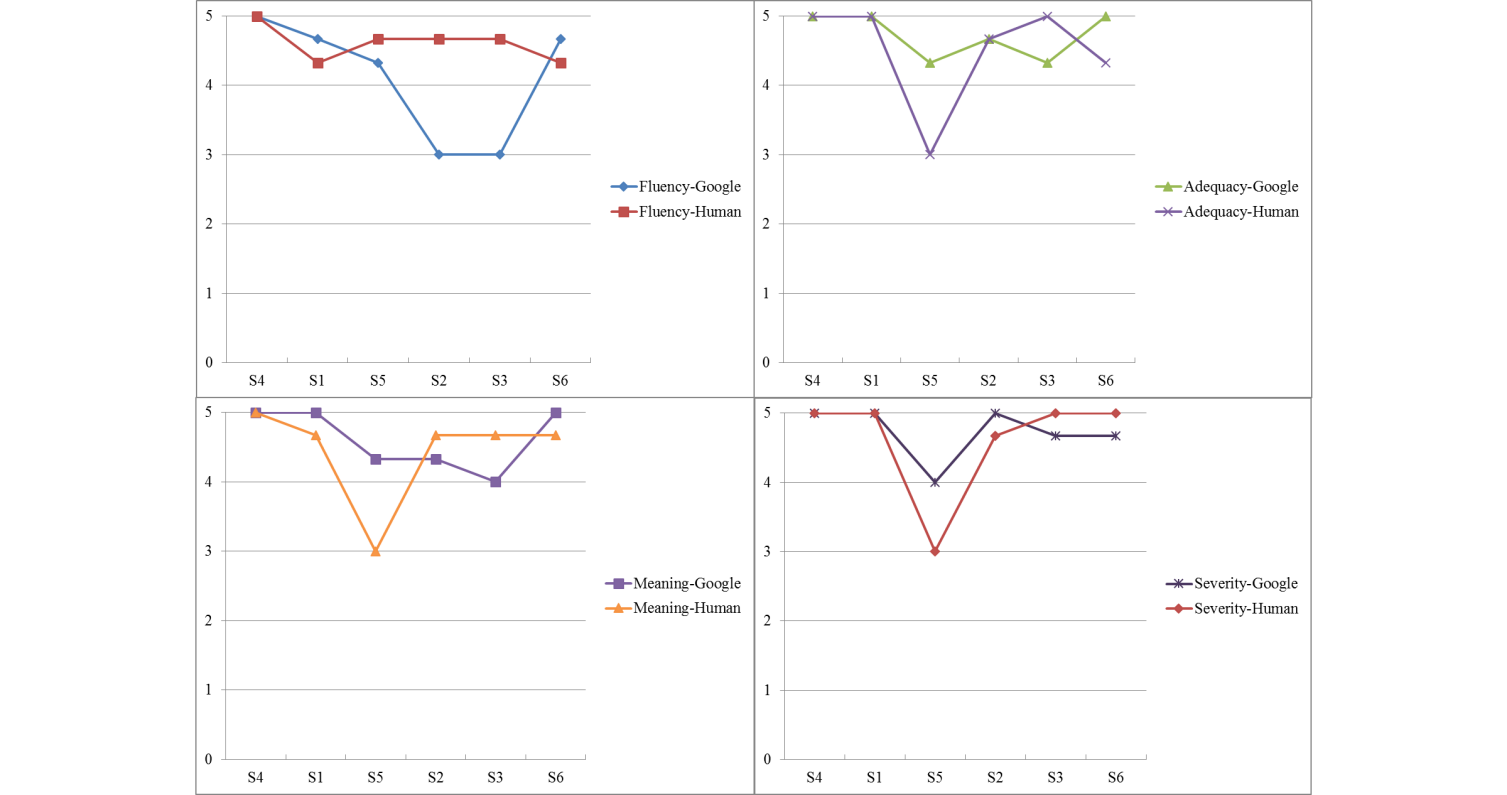
Spanish Google versus human.

**Figure 3:**
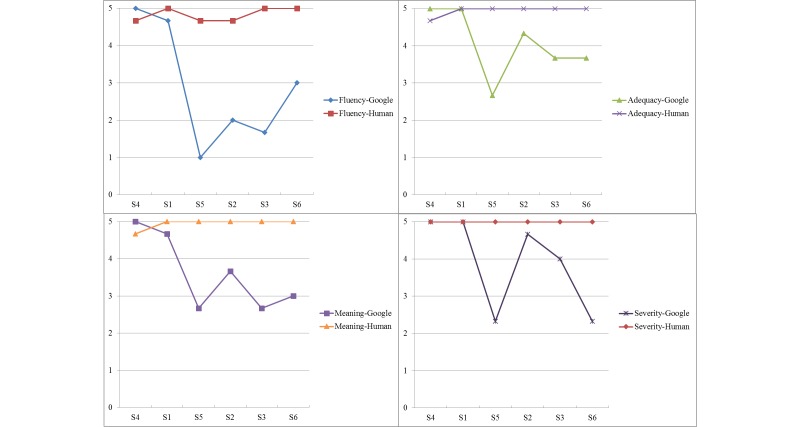
Chinese Google versus human.

As shown in Figures 2 and 3, to better compare and capture the trends among sentences with regard to the accuracy scores on four domains, we ranked the sentences according to their grade levels—presenting the easiest sentence (S4) first and the most difficult sentence (S6) last. As shown in Figure 2, when sentences were translated from English to Spanish, S2 and S3 (more difficult sentences) had a considerable difference between Google and human in the Fluency domain, where the human translator did much better than Google. For the relatively easy sentences (S4 and S1), there was not much difference between Google and human in any of the four domains. Interestingly, there was not much difference for the most difficult sentence (S6) either. We also noticed some obvious gaps for S5 (medium difficult sentence) in the Adequacy, Meaning, and Severity domains, where Google received a higher translation accuracy (English to Spanish) than the human translator did. As shown in Figure 3, when sentences were translated from English to Chinese, S5, S2, S3, and S6 (more difficult sentences) had a considerable difference between Google and human in all four domains, where the human did much better than Google (except S2 in the Severity domain). Similar to what we found in the Spanish set, there was not much difference between Google and human in all domains for the easier sentences (S4 and S1). When comparing between Figures 2 and 3, results showed that the general distance between Google and human for Chinese is larger than Spanish, indicating that Google provided higher accuracy translation service in Spanish than in Chinese.

## Discussion

### Principal Findings

This pilot study evaluated the accuracy of Google Translate when translating diabetes patient education materials from English to Spanish and English to Chinese. We found that Google provided accurate translation for simple sentences, but the likelihood of incorrect translation increased when the original English sentences required higher grade levels to comprehend. For example, the most simple sentence in our study (“Stop smoking”) translated by Google received full scores on every domain for both languages, while Google received lower scores on more difficult sentences (eg, S5 and S6) for both languages. The Chinese human translator provided much more accurate translation than Google did. The Spanish human translator, on the other hand, did not provide a significantly better translation compared to Google. Additionally, we identified some sentences translated by Google from English to Chinese that might lead to delayed patient care. Similarly, one sentence translated by the professional human translator from English to Spanish could also have a negative impact on patients. The results demonstrate that Google is capable of producing a more accurate translation from English to Spanish than English to Chinese.

Google provided more accurate translations for sentences with lower readability levels but made more translation errors on sentences with higher readability levels, especially when translating sentences from English to Chinese. Although we did not find any statistically significant correlation between readability and translation accuracy due to our small sample size, our findings seem to be consistent with previous investigations that document a significant negative correlation between sentence length and translation accuracy (*r*=-.4393, *P*<.05), indicating that the machine was less likely to provide correct translation for longer sentences [27].

Google yielded high error rates when translating English sentences to Chinese. We identified several problematic sentences. S2 and S3 translated by Google from English to Chinese had marginal fluency with several grammatical errors, but the evaluators were able to make enough sense of them to get a meaning close to the original sentences. Thus, these two sentences did not have much negative impact on patient care. However, S5 (“Take medicines the way your doctor tells you”) translated by Google from English to Chinese had no fluency and was not understandable. After translation, this sentence in Chinese did not make sense to the evaluators. Therefore, this sentence might cause delayed patient care. Likewise, S6 (“Ask your doctor about taking medicine to protect your heart, such as aspirin or a statin”) translated by Google from English to Chinese added misleading information into the original sentence by translating it as “Ask your doctor about taking Chinese medicine to protect your heart, such as aspirin or a statin.”

Compared to Chinese, Google provided noticeably higher accuracy when translating sentences from English to Spanish. All the Spanish sentences conveyed more than 75% of the original information and had almost the same meaning as the original sentences. Moreover, none of them had a severe impact on patient care (Severity≥4.67). Consistent with our findings, Zeng-Treitler et al [27] also found that Spanish machine translation had higher accuracy than other languages: Spanish had 33.8% correctness compared to the correctness of Chinese, Russian, and Korean, which ranged from 7.98% to 11.74%. Zeng-Treitler et al [27] contend that “one possible explanation for this may well lie in the fact that English and Spanish are more similar (eg, word order, inflections) than English and Chinese, Korean or Russian” (p. 76).

The Chinese human translator provided much more accurate translation than Google; however, the Spanish human translator did not provide a significantly better translation than Google. In contrast to our findings, Khanna et al [17] reported that Google made more errors than human translators when translating patient education materials from English to Spanish. Zeng-Treitler et al [27] concluded that Babelfish was not a good machine translation tool because of its high percentage of inaccuracy.

We identified one problematic sentence (S5 “Take medicines the way your doctor tells you”); the translation by the Spanish human translator might cause delayed patient care. This sentence was also problematic when translated by Google from English to Chinese. It conveyed half of the original information and partially the same meaning as the original sentence. The Spanish human translator twisted the meaning of the original English sentence by creating a Spanish sentence saying “Tome las medicinas recetadas por su médico,” meaning “Take the medicine prescribed by your doctor.” Such incorrect translation provided by the Spanish human translator might lead to delayed necessary patient care.

We also wish to highlight that in some cases professional human translators might also make severe errors that negatively impact patients’ health compared to machine translation tools. Flores et al [28] contend that the most common types of mistake by human interpreters, which could potentially cause medical accidents, include omission, false fluency, substitution, editorialization, and addition. For this reason, we recommend continuous training and credential practice standards for professional medical translators to enhance patient safety. For example, Michael et al [29] developed a translation standard to guide the language-translating process for health education information (see Textbox 1) with 10 key components (p. 550).

**Translation standard with 10 key components.**

1. Develop the English text and/or test the translation with members of the target LOTE (a language other than English)-speaking community.

2. Undertake a cultural and linguistic assessment of the English text in preparation for its translation.

3. Undertake a subject matter expert assessment of the English text as appropriate.

4. Organize for the English text to be translated by a professional translator.

5. Undertake a cultural and linguistic assessment of the translation.

6. Organize for the translation to be proofread by a professional translator.

7. Include the title of the text in English on the translation.

8. Include the name of the target language in English, on both the English text and the translation.

9. Distribute the translation in bilingual format—English and LOTE.

10. Date, monitor, evaluate, and update the English text and the translation as part of an ongoing review program.

In addition to ensuring human translation accuracy, improvements to machine translation tools are also necessary prior to use by patients and health care providers. Health educators should make efforts to achieve higher translation accuracy for machine tools and ultimately make sure health education information is not misinterpreted and necessary care not delayed. Mismatches between the vocabulary bank in machine translation systems and the terminologies used in the original language texts are common sources of machine translation errors [30]. Developing a universal code system for machine translation can improve language translation accuracy [31]. Therefore, we call for collaborations between computer science engineers and public health/health education professionals to work on this language translation technique, which could assist LEP populations better understand health information.

Furthermore, health education information should be written in multiple languages other than English and Spanish. In one study, Becker [1] examined 125 websites that provided health information in the United States and reported that only 10% of the state sites provided Spanish versions. Moreover, these Spanish webpages contained many English texts such as Web link buttons labeled in English. Most health institutions do not provide information in multiple languages besides English on their websites, but Internet users prefer searching for health information using local languages instead of English. Immigrants in particular prefer seeking and reading health information in their native languages rather than the languages of the adopted country [32].

### Limitations

Our study has three limitations that should be noted. First, we recruited ATA-certified translators as evaluators who, because of their professional training, had more credibility for scientifically evaluating translation accuracy than non-professional bilinguals such as graduate students. Translators also have different translation styles and knowledge of second language audiences. The selection of certified translators might cause measurement bias because these professional translators are different from general LEP patients. For instance, compared to LEP patients, certified translators are bilingual, well-educated, and have higher literacy levels. Thus, sentences that are understandable to them might not be understandable to LEP patients. Future research might recruit LEP participants to evaluate these translation products, and researchers might conduct cognitive interviews while participants read these sentences. Second, our study mainly focused on describing the translated products from a technical perspective instead of assessing message consumers’ experience from a user perspective. Testing LEP diabetes patients’ knowledge and behavior change after using Google Translate to process health education messages is another direction for future study. Finally, our study sample size was small. We evaluated six original English sentences and recruited 6 evaluators, which had less power for generalizability. Researchers should include a large sample of original sentences and evaluators for future study.

### Conclusions

Notwithstanding these limitations, this investigation provides important contributions to the ever-growing literature base examining the effectiveness of machine translation tools. In particular, our findings highlight that as sentences become more complex in health information and require higher levels of reading ability, the likelihood of machine translation tools making errors increases. As shown in the paper, these errors have the potential to negatively impact patient health behaviors. Given that medical or health advice is not always delivered in short, easy-to-understand sentences, such as those at a 2.8 grade reading level (eg, “Stop smoking”), it is imperative that future investigations continue to examine the real-world application of machine translation tools and their associated impact on patient and population health.

## Abbreviations

ATA: American Translators Association
LEP: limited English proficiency
LOTE: language other than English
MANOVA: multivariate analysis of variance
